# Emotional Intelligence in Gen Z Teaching Undergraduates: The Impact of Physical Activity and Biopsychosocial Factors

**DOI:** 10.3390/ejihpe15070123

**Published:** 2025-07-04

**Authors:** Daniel Sanz-Martín, Rafael Francisco Caracuel-Cáliz, José Manuel Alonso-Vargas, Irwin A. Ramírez-Granizo

**Affiliations:** 1 Faculty of Humanities and Social Sciences, Universidad Isabel I (Ui1), 09003 Burgos, Spain; 2 Faculty of Health Sciences and Education, Distance University of Madrid (UDIMA), 28010 Madrid, Spain; rafaelfrancisco.caracuel@udima.es; 3 Faculty of Education Sciences and Humanities, International University of La Rioja (UNIR), 26006 Logroño, Spain; 4 Faculty of Education Sciences, International University of Valencia (VIU), 46002 Valencia, Spain; 5 Department of Didactics Musical, Plastic and Corporal Expression, Faculty of Education Science, University of Granada, 18071 Granada, Spain; alonsojm@ugr.es (J.M.A.-V.); irwinrg@ugr.es (I.A.R.-G.)

**Keywords:** emotional attention, emotional clarity, emotional repair, moderate–vigorous physical activity, social networks, university, Spain, health, addiction, sex

## Abstract

Emotional intelligence is a crucial determinant of socioemotional adaptation, psychological well-being and healthy habits in a population, although it has been barely studied in Generation Z. Therefore, the following research objectives were established: (1) to measure the levels of attention, clarity and emotional repair of Spanish university students in teaching undergraduates and (2) to design predictive models of emotional intelligence considering sex, anthropometric measurements, physical activity and the use of social networks as factors. A cross-sectional study was conducted with the involvement of Spanish teaching undergraduates. An online questionnaire integrating sociodemographic questions, the International Physical Activity Questionnaire Short Form, Trait Meta-State Mood Scale TMMS-24 and Social Network Addiction Scale SNAddS-6S were administered. University students exhibited higher levels of emotional attention (30.32 ± 6.08) than those of emotional clarity (28.18 ± 6.34) and emotional repair (28.51 ± 6.02). Most students use X, Pinterest, TikTok, Instagram, YouTube and WhatsApp most days of the week. There are positive relationships between attention and emotional clarity (r = 0.33; *p* ≤ 0.001), attention and emotional repair (r = 0.18; *p* ≤ 0.001) and clarity and emotional repair (r = 0.44; *p* ≤ 0.001). In conclusion, males have higher levels of emotional clarity and emotional repair, but females show higher levels of emotional attention. The model with the highest explanatory power is the one obtained for men’s emotional attention.

## 1. Introduction

Emotional intelligence is ‘the subset of social intelligence that involves the ability to monitor one’s own and others’ feelings and emotions, to distinguish between them, and to use this information to guide thinking and actions’ (Salovey & Mayer, 1990, p. 89) and integrates three dimensions: attention, clarity and emotional repair (Fernández-Berrocal et al., 2004). This type of intelligence is related to personal, family and social problems that are caused by poor emotional management (Flores, 2023), plays a special role in personality formation (Renom, 2008) and affects any decision that people make (Sánchez-Bolivar et al., 2023). In this context, studies of emotional intelligence levels in young adults and the factors that cause them currently have significant implications because this group suffers from high levels of anxiety, depression and stress, which are higher than those of other age groups such as adults (Bear et al., 2021).

The levels of attention, clarity and emotional repair are not similar among Spanish university students. On the one hand, Acebes-Sánchez et al. (2019) identify higher levels of emotional attention than emotional repair and, in turn, higher than those of emotional clarity. On the other hand, Gavín-Chocano et al. (2020) demonstrated that students who are teaching undergraduates in Granada and Jaén (Spain) had higher levels of emotional clarity compared to those of emotional attention and both were also higher compared to those of emotional repair.

The emotional intelligence levels of university students are influenced by various biological, psychological and lifestyle factors. Sex is one of these factors of emotional intelligence, and although there is no clear trend according to sex, most studies state that men have higher levels than women (Cañero et al., 2019; Ubago-Jiménez et al., 2021). In contrast, stress has a negative relationship with emotional intelligence (Sepdanius et al., 2023). In addition, light, moderate and vigorous physical activity are also positively associated with emotional intelligence (Ubago-Jiménez et al., 2021). Likewise, emotional intelligence is also positively related to job satisfaction and happiness in Spanish teachers of children and adolescents (García-Domingo & Quintanal, 2022).

The study of the relationship between emotional intelligence and addiction is an emerging area in the current scientific literature (Villarreal et al., 2020), although existing evidence is still very limited (Henning et al., 2021). In this context, emotional intelligence appears to be negatively associated with drug consumption (Villarreal et al., 2020) and alcohol use (Villarreal-Mata et al., 2024). Similarly, social network addiction appears to be negatively related to emotional intelligence (Jarrar et al., 2022), although this relationship varies depending on the intelligence dimension. For instance, Rivera-Véliz and Araujo-Robles (2020) concluded that there was a positive relationship between emotional attention and the lack of control in the use of social networks and between emotional attention and the excessive use of social networks; in contrast, the relationship was negative between emotional clarity and social network obsession and between emotional repair and social network obsession.

Another factor that appears to influence emotional intelligence is the use of social networks, although more research is needed (Arrivillaga et al., 2022). Social networks are ‘places on the Internet where people publish and share all kinds of personal and professional information with third parties, acquaintances and absolute strangers’ (Celaya, 2008, p. 92). Some of the most popular social networks are Facebook, Telegram, Instagram, TikTok, Pinterest, X (formerly Twitter), WhatsApp, Snapchat, YouTube, Discord and Twitch (Chaffey, 2023; Newman et al., 2023; Sarman & Tuncay, 2023).

Over sixty percent of the global population, equivalent to 4.89 billion people, use social networks, spending an average of 1 h and 24 min per day (Chaffey, 2023), although these levels vary according to age and sex (Chaffey, 2023; Newman et al., 2023). These levels are higher in Spain, where 85.6% of the population has social networks (Forner, 2023). The levels of young people (15–26 years old) are even more alarming, spending on average 5 h and 30 min using social networks (Fernández-Rovira, 2022). Such is the use of social networks that 20% of the population in eastern and southern Europe can be considered addicted (Cheng et al., 2021) to the extent that young people from Generation Z report feeling insecure, angry and/or sad if their posts do not receive the response they expect (likes or comments) (Martín & Medina, 2021). In addition, Spanish women have higher levels of social network addition (Cuadrado et al., 2020). Faced with this problem, the WHO (2024) considers taking measures to prevent the negative impact of technology on the well-being and mental health of citizens as a current priority. In this context, most studies on social networks have described levels of the use of certain social networks like X, Facebook, Instagram and TikTok (Fernández-Rovira, 2022), but few have analyzed the influence of personal factors (Kircaburun & Griffiths, 2018).

Due to the importance of emotional intelligence in personal development and competence today (Renom, 2008; Sánchez-Bolivar et al., 2023), the current study focused on measuring emotional intelligence levels of Spanish teaching undergraduates among Generation Z and their relationship with physical activity and other biopsychosocial determinants. In this way, proposals could be designed to improve the emotional intelligence levels of university students more accurately and effectively, improving their training and counseling needs for future teachers (Dans et al., 2021). This research had the following aims:

(1) To measure the levels of attention, clarity and emotional repair of Spanish teaching undergraduates.

(2) To design predictive models of emotional intelligence considering sex, anthropometric measurements, physical activity and the use of social networks as factors.

Based on the above aims, the following research hypotheses were established:

**H1.**  *The emotional intelligence levels of males are higher than those of females*.

**H2.**  *The levels of emotional attention are higher than those of emotional clarity and both are higher than those of emotional repair*.

**H3.**  *There are valid sex-different models predicting emotional attention, clarity and repair*.

## 2. Materials and Methods

### 2.1. Design and Participants

A descriptive correlational cross-sectional study was conducted to examine the levels of emotional intelligence and the biological, psychological and social factors that affect them in Spanish teaching undergraduates who are part of Generation Z (born between 1995 and 2010) (Cortés et al., 2023). The Ethics Committee 3132/CEIH/2023 of the University of Granada approved the conduct of the study (date of approval 19 January 2023). In addition, the ethical principles of the Declaration of Helsinki were followed. The study was conducted between October 2023 and February 2024.

The study population was composed of teaching undergraduates and consisted of 150,565 students for the academic year 2022–2023 (MEVT, 2023). The research questionnaire was sent by e-mail to the management of all the Spanish faculties and universities offering teaching degrees so that it could be forwarded to the students. Ultimately, 423 students took part, 12 of them being excluded because they did not belong to Generation Z. Thus, the final sample was 411 young people and representative of the population under study (assuming a standard deviation of 50 and a 95% variance, implying an estimation error of 4.83%).

The mean age of the participants was 20.47 years (±1.89) and 68.86% (*n* = 283) were women. Additionally, 22 Spanish provinces (44.31%) and 11 autonomous communities (64.71%) were represented.

### 2.2. Instrument and Variables

Participants responded without reimbursement to a Google^®^ Forms questionnaire (Mountain View, CA, USA) that had three main sections.

#### 2.2.1. Sociodemographic Section

An ad hoc design was used, where participants were asked about their sex, age, Spanish region of residence, weight and height. Body mass index (kg/m^2^) was also calculated (WHO, 2021). This section asked about the availability and use of social networks as well. Two variables were established from the responses: the number of social networks where participants have an account and the number of social networks they use most days of the week (4–7 days).

#### 2.2.2. Physical Activity Level Section

This section used the International Physical Activity Questionnaire Short Form (IPAQ-SF), which was adapted for Spanish undergraduates and validated by comparing the results with those of a pedometer (r = 0.51; *p* ≤ 0.05), with a uniaxial accelerometer (r = 0.47; *p* ≤ 0.05) and with a triaxial accelerometer (r = 0.49; *p* ≤ 0.05) (Rodríguez-Muñoz et al., 2017). The IPAQ-SF has previously been used in other studies, such as those carried out by López-Olivares et al. (2020) and Ramón-Arbués et al. (2022).

The questionnaire comprises seven questions on physical activity levels during a typical week, with the first two relating the number of days and average daily time spent performing vigorous physical activity and the next two similar for moderate intensity physical activity. Questions five and six are about the number of days and the average daily walking time, while question seven is about the average daily sitting time on working days. This study identified three variables expressed in minutes per week: time spent performing vigorous physical activity, time spent performing moderate physical activity, and time spent performing moderate-to-vigorous physical activity.

#### 2.2.3. Emotional Intelligence Level Section

The Trait Meta-State Mood Scale (TMMS-24) was used in the version validated by Fernández-Berrocal et al. (2004) for Spanish adults. TMMS-24 showed internal consistency scores above α = 0.85, and the correlations between the pre-test and post-test scores were reliable (emotional attention: r = 0.60; emotional clarity: r = 0.70; emotional repair: r = 0.83) (Fernández-Berrocal et al., 2004). This instrument has previously been used by Carbonero-Martín et al. (2022) and Redondo-Rodríguez et al. (2023). TMMS-24 integrates 24 questions on a 5-point Likert scale (1 ‘strongly disagree’ and 5 ‘strongly agree’) on three dimensions of emotional intelligence (attention, clarity and repair), each dimension comprising eight questions and with a final score between 8 and 40 points. The score for each dimension of emotional intelligence is calculated by assigning one point for each Likert scale value.

The dimension of emotional attention includes questions such as ‘Do I pay a lot of attention to my feelings?’ and ‘Do I usually spend time thinking about my emotions?’ In turn, the emotional clarity dimension includes questions such as ‘Am I clear about my feelings?’ and ‘Do I almost always know how I feel?’ Finally, some examples of questions in the emotional repair dimension are ‘Do I care about having a good mood?’ and ‘Do I have a lot of energy when I feel happy?’

Three separate variables were analyzed, each representing a dimension of emotional intelligence, and their internal consistencies were strong (emotional attention: α = 0.874; emotional clarity: α = 0.913; emotional repair: α = 0.849).

#### 2.2.4. Social Network Addiction Section

This section comprised the social network addiction scale (SNAddS-6S), which was designed and validated for Spanish adults by Cuadrado et al. (2020) and previously used by López-Gil et al. (2023) and Gallegos and Flores (2021). This scale is useful for assessing individuals’ addiction levels and identifying predictors of addiction (Cuadrado et al., 2020). The scale was initially validated with an α reliability of 0.89, and an optimal exploratory factorial analysis (Kaiser–Meyer–Olkin index = 0.80; Bartlett’s test of sphericity: χ^2^ = 1612.74; df = 153; *p* < 0.001) was performed, explaining 73.38% of the variance and Confirmatory Factor Analysis (χ^2^ (df = 5) = 2.689; *p* < 0.611; χ^2^/DF = 0.67; the root-mean-square error of approximation = 0.001; the comparative fit index = 1.00; the goodness-of-fit index = 1.00; the adjusted goodness-of-fit index = 0.98; the Akaike information criterion = 36.69) (Cuadrado et al., 2020).

The SNAddS-6S includes 18 questions covering five dimensions: the first 6 items cover time management, the next 3 comprise mood modification, the following 3 cover relapse, items 13–15 focus on withdrawal and the last 3 cover conflict. An example of an item from each of the five dimensions is as follows: ‘Have you spent a lot of time thinking about or planning to use social networks?’, ‘Have you used social network to reduce feelings of guilt, anxiety, helplessness and/or depression?’, ‘Have others advised you to reduce your use of social network, but you did not listen?’, ‘Have you become restless or worried if you have been banned from using social network?’ and ‘Have you used social network so much that it has had a negative impact on your work or studies?’ Responses are given on a Likert scale from 1 to 5 (1 being ‘never’ and 5 being ‘very often’).

In this study, one variable was identified for each dimension of social network addiction, obtaining the following internal consistencies: α = 0.820 for time management, α = 0.910 for mood modification, α = 0.803 for relapse, α = 0.867 for withdrawal and α = 0.777 for conflict.

### 2.3. Data Analysis

IBM SPSS 26.0 software (International Business Machines Corporation, Armonk, NY, USA) was used for the statistical analysis and it was divided into two phases: (1) a descriptive and correlational phase for all the variables and (2) a predictive phase for the levels of each dimension of emotional intelligence. In the descriptive phase of the statistical analysis, mean values and standard deviations were calculated for age, weight, height, body mass index, the number of social networks, the number of social networks used most days of the week (4–7 days), emotional attention, emotional clarity, emotional repair, time management social network addiction, mood modification social network addiction, relapse social network addiction, withdrawal social network addiction, conflict social network addiction, moderate physical activity, heavy physical activity and moderate-to-heavy physical activity. For this purpose, mean values were shown for all participants and according to sex (male or female), calculating the difference in means according to sex by applying Student’s *t*-tests for independent samples and Cohen’s *d* values to determine the effect size.

The descriptive phase of the statistical analysis also describes the number and percentage of participants using the different social networks most days of the week (4–7 days). Chi-square and Cramer’s V tests were applied to identify differences in the use of social networks according to the sex of the participants. Additionally, Pearson’s correlation test was used to analyze the relationship between continuous research variables.

In the predictive part of the statistical analysis, multiple linear regression models predicting levels of emotional attention, clarity and repair were validated because they met the following requirements: (1) pre-model linearity between the dependent and independent variables (Pearson correlation), (2) the independence of the model residuals (Durbin–Watson), (3) homoscedasticity (scatter plot and residual errors), (4) the normal distribution of the errors (the Kolmogorov–Smirnov test) and (5) non-multicollinearity between the independent variables (VIF). The regression models were identified using the stepwise method.

## 3. Results

The participants’ ages ranged from 18 to 28 years, with an average age of 20.47 years (SD = 1.89), being significantly higher in males than in females (20.91 ± 1.76 years and 20.28 ± 1.92 years, respectively, t = 3.15; *p* = 0.002; d = 0.34). In addition, men had significantly higher weight, height and BMI values than women (*p* ≤ 0.001).

Regarding emotional intelligence, men’s mean score for emotional attention was lower than women’s (t = −4.94; *p* ≤ 0.001; d = 0.52), while men had higher levels of emotional clarity and emotional repair, the latter being significantly higher (t = 3.59; *p* ≤ 0.001; d = 0.38).

In relation to the social network addiction factors, females scored higher than males in all of them, except in conflict social network addiction, without significant differences between the mean scores.

The mean time spent by men in vigorous physical activity was 401.05 ± 329.75 min/week, compared to 220.60 ± 305.73 min/week for women, with a difference between these mean values (*p* ≤ 0.011; d = 0.64). Similarly, males also obtained higher mean scores for moderate–vigorous physical activity levels (574.45 ± 443.04 and 334.84 ± 455.93, respectively; *p* ≤ 0.001; d = 0.53).

Table 1 shows the means and standard deviations of the continuous research variables for all participants and according to sex. In addition, Tables S1–S3 show the correlations between all these study variables for the whole sample of participants, for males and females, respectively.

Table 2 shows the frequencies of social network use on most days of the week (4–7 days) according to the sex of the participants. There are significant differences in the use of five social networks: Snapchat (x^2^ = 11.52, *p* ≤ 0.001; V = 0.17), Telegram (x^2^ = 20.41, *p* ≤ 0.001; V = 0.22), Pinterest (x^2^ = 94.29, *p* ≤ 0.01; V = −0.48), Twitch (x^2^ = 71.24, *p* ≤ 0.001; V = 0.42) and Discord (x^2^ = 7.32, *p* = 0.007; V = 0.13). This also highlights that all participants use WhatsApp most days.

Table 3 presents the statistics of the multiple linear regression models for predicting attention, clarity and emotional repair for all participants. Regarding the emotional attention regression model, it accounted for 28.3% of the variance. The final model obtained was as follows: emotional attention = 10.409 + 0.338 * emotional clarity + 0.443 * mood modification social network addiction + 3.357 * sex + 0.108 * emotional repair + 0.129 * time management social network addiction − 1.189 * X use. Moreover, positive relationships of emotional attention with time management social network addiction (r = 0.23; *p* ≤ 0.001), mood modification social network addiction (r = 0.27; *p* ≤ 0.001), emotional clarity (r = 0.33; *p* ≤ 0.001), emotional repair (r = 0.18; *p* ≤ 0.001) and negative relationship with X use (r = −0.11; *p* = 0.03) were observed.

The model obtained for emotional clarity was as follows: emotional clarity = 10.677 + 0.373 * emotional repair + 0.358 * emotional attention − 0.287 * relapse social network addiction − 0.286 * conflict social network addiction − 1.361 * sex + 1.323 * X use. In addition, emotional clarity and emotional repair are positively associated (r = 0.44, *p* ≤ 0.001), as are emotional clarity and attention (r = 0.33, *p* ≤ 0.001), but negatively associated with relapse social network addiction (r = −0.15, *p* = 0.002) and conflict social network addiction (r = −0.15, *p* = 0.002).

Regarding emotional repair, the model obtained was as follows: emotional repair = 17.119 + 0.364 * emotional clarity − 2.062 * sex − 0.388 * mood modification social network addiction + 0.308 * conflict social network addiction + 0.115 * emotional attention. Similarly, there are significant correlations between emotional repair and emotional clarity (r = 0.44; *p* ≤ 0.001), sex (r = −0.18; *p* ≤ 0.001), mood modification social network addiction (r = −0.15; *p* = 0.002) and emotional attention (r = 0.18; *p* ≤ 0.001).

The statistics of the regression models for men’s emotional attention, clarity and repair are presented in Table 4. The model obtained for men’s emotional attention was as follows: 12.039 + 0.680 * mood modification social network addiction + 0.345 * emotional clarity − 2.259 * X use + 2.407 * Facebook use − 7.106 * Instagram use + 0.535 * withdrawal social network addiction − 0.480 * conflict social network addiction. This model is useful for explaining 35.4% of the variance. In addition, significant correlations were observed between emotional attention and mood modification social network addiction (r = 0.32; *p* ≤ 0.001), emotional clarity (r = 0.30; *p* ≤ 0.001), X use (r = −0.27; *p* = 0.002), Instagram use (r = −0.21; *p* = 0.016) and conflict social network addiction (r = 0.21; *p* = 0.017).

The emotional clarity model for men accounted for 33.7% of the variance and was as follows: emotional clarity = 13.423 + 0.383 * emotional repair − 12.736 * YouTube use − 0.566 * relapse social network addiction + 0.233 * emotional attention + 0.003 * vigorous physical activity. In addition, there are significant correlations between emotional clarity and emotional repair (r = 0.39; *p* ≤ 0.001), YouTube use (r = −0.28; *p* = 0.002), relapse social network addiction (r = −0.20; *p* = 0.025) and emotional attention (r = 0.30; *p* ≤ 0.001).

In relation to the emotional repair model for men, this accounted for 18.4% of the variance and was as follows: emotional repair = 22.916 + 0.383 * emotional clarity − 0.992 * social networks use.

Table 5 includes the statistics of the regression models for women’s emotional attention, clarity and emotional repair. The model obtained for women’s emotional attention was 15.433 + 0.320 * emotional clarity + 0.482 * mood modification social network addiction + 0.117 * emotional repair. This model is useful for explaining 22.1% of the variance. In addition, emotional attention and emotional clarity (r = 0.39; *p* ≤ 0.001), emotional attention and mood modification social network addiction (r = 0.23; *p* ≤ 0.001) and emotional attention and emotional repair (r = 0.25; *p* ≤ 0.001) are positively linked.

Regarding women’s emotional clarity model, it accounts for 32.4% of the variance and is as follows: 7.577 + 0.379 * emotional repair + 0.362 * emotional attention − 0.339 * relapse social network addiction + 2.225 * X use. Similarly, significant correlations were found between emotional clarity and emotional repair (r = 0.44; *p* ≤ 0.001), relapse social network addiction (r = −0.13; *p* = 0.033) and X use (r = 0.17; *p* = 0.004).

Finally, the model related to the emotional repair of women only includes one variable that accounts for 19.4% of the variance. The regression model is as follows: emotional repair = 16.145 + 0.420 * emotional clarity.

## 4. Discussion

The present research had the following objectives: (1) to measure the levels of attention, clarity and emotional repair of Spanish teaching undergraduates and (2) to design predictive models of emotional intelligence considering sex, anthropometric measurements, physical activity and social network use as factors.

Although the emotional intelligence levels of Generation Z university students in Spain are high (with scores close to 30 points in an interval of 8–40), they could be better. The total score (87.06) is higher than those obtained by Acebes-Sánchez et al. (2019) and Cañero et al. (2019), which were 85.3 and 79.41, respectively.

A significant difference was obtained in emotional attention, with higher levels in women, and emotional repair, which was in favor of men. Additionally, men had higher levels of emotional clarity, though this difference was not significant. These findings are similar to those of Acebes-Sánchez et al. (2019) and Ubago-Jiménez et al. (2021), who also found superiority in emotional intelligence as a function of sex. However, in both of these studies, all differences were statistically significant.

No significant differences between the sexes in social network use or addiction were found. Despite this, women have accounts on more social networks and men use them more on most days of the week. In addition, women show higher levels of social network addition in most dimensions except for conflict social network addiction, which is slightly higher in men. Consistent with these findings, Lozano et al. (2020) and Xu et al. (2022) also showed that women had higher levels of social network addiction.

Women use Snapchat and Pinterest more frequently than men, who use Telegram and Twitch more often. WhatsApp is the most used social network by undergraduates most days of the week (100%), but others are also used by most young people: Instagram (98.54%), YouTube (97.32%), TikTok (85.40%), X (72.02%), and Pinterest (60.58%).

These results differ from those of Chaffey (2023), who found that Facebook and YouTube were the most popular platforms. Similarly, Forner (2023) found that Spanish women preferred using Facebook, YouTube, Instagram and TikTok. The differences in the use of social networks may be due to the purposes for which young people use them, whether as a means of communication, publishing files, researching academic topics or for leisure (Fernández et al., 2020), because women may use them for editing and posting videos and photos and for fashion, which are connected with the use of Facebook, Instagram and TikTok (Forner, 2023; Pacheco, 2021).

Spanish university students studying teaching degrees and belonging to Generation Z have mean moderate–vigorous physical activity levels (409.46 ± 464.88 min/week) that are higher than those recommended by the WHO (2020) for adults (180 min/week). The mean levels of women are almost double the recommended levels and those of men more than triple, with significant differences depending on sex. Likewise, there are also differences in the levels of vigorous physical activity, with men’s being more than double that of women. Moderate and vigorous physical activity levels are lower and higher, respectively, than those obtained by Corella et al. (2018) in Spanish undergraduates. Moreover, moderate and vigorous physical activity levels in this study are lower than those in the study by Romero-Blanco et al. (2020).

Every multiple linear regression model that was developed is valid for predicting the levels of emotional attention, clarity and repair, with different predictors depending on the sex of the participants. The model with the highest predictive value is that relating to men’s emotional attention, accounting for 35.4% of the variance. In addition, the three identified models of emotional clarity predict more than 31% of the variance. The most predominant predictor variables of the models are the dimensions of emotional intelligence different to the model itself and mood modification social network addiction, although there are also other variables of addiction and social network use that are influential. Additionally, the influence of physical activity in the models is very limited, being reduced exclusively to vigorous physical activity in relation to emotional clarity.

The different dimensions of emotional intelligence are predominantly positively and significantly connected with each other. In addition, the use of some social networks is negatively related to the dimensions of emotional intelligence, such as the use of X (former Twitter). This is related to the findings of Süral et al. (2018) who identified a model for adults in which emotional intelligence was negatively linked to problematic social network use.

Although the obtained multiple regression models are valid, a high percentage of the variance of emotional intelligence still remains unexplained. Therefore, there could be other predictors of emotional intelligence not considered in this study, such as the academic year period, emotional exhaustion (Sánchez-Pujalte et al., 2021), subjective well-being (Zhao, 2021) and stress (Sepdanius et al., 2023).

This research carried out with Spanish undergraduates has three main limitations. First, the participants were teaching undergraduates who received the link to the questionnaire through the forwarding of the questionnaire by the university directors, who decided whether to forward it or not, limiting the access to the participants. To address this limitation, the invitation e-mail was sent to all Spanish university heads and we accepted participants who responded correctly. In this way, a nationally representative sample was obtained, covering most of the Spanish regions.

Secondly, the instrument administered was an online questionnaire, which is a limitation itself. This instrument made it possible to collect information from many participants from different regions without having to make an economic outlay, but it also implies that there was no researcher present in person during the response time, although an e-mail address was available for queries. In addition, the physical activity questionnaire, although designed and validated for the study population, is much less accurate than other instruments such as accelerometers. This limitation is also evident in the way the participants’ emotional intelligence levels were assessed as a self-reported questionnaire was used. To address this drawback, an instrument adapted and validated for use with young Spanish people was used, and its internal consistency was found to be greater than α = 0.849, meaning the results obtained are valid.

Thirdly, the cross-sectional nature of the study introduces limitations. This means that while it is possible to identify relationships between the variables included in this manuscript, it is not possible to make causal inferences that would allow us to determine the directionality of these relationships.

Some future lines of research have been identified from the results obtained. For example, it would be useful to carry out a longitudinal study to identify whether the levels of emotional intelligence vary according to the period of the academic year and in different years. In addition, randomized controlled trials could also be conducted to identify causal inferences between variables, for example, between social network use and levels of emotional intelligence. Another proposal is to compare the levels of the different variables analyzed with those of other population groups such as millennials. Likewise, it would be possible to analyze the qualitative influence of the use of the most important social networks on the levels of emotional intelligence. In the same vein, it would be appropriate to design similar studies including other biopsychosocial variables to try to identify other predictors that explain more variance in the regression models. Finally, it is recommended to design actions to improve the levels of emotional intelligence, although those obtained are not excessively low. These interventions should cover the three dimensions of emotional intelligence, since they are interrelated, and prioritize emotional clarity and repair since the scores obtained for these were lower than those for emotional attention. In addition, this would also be indirectly beneficial in reducing the levels of addiction to social networks (Arrivillaga et al., 2022).

## 5. Conclusions

Males have higher levels of emotional clarity and emotional repair, but females show higher levels of emotional attention. Among the whole sample and females specifically, emotional attention and emotional repair levels are higher than those of emotional clarity. In contrast, emotional repair is higher than emotional clarity and both with respect to attention for males. In this context, emotional intelligence could vary significantly across the lifespan, meaning these conclusions may have limited applicability in general.

The predictive models obtained are valid for each of the dimensions of emotional intelligence. These models predict different percentages of the variance depending on the dimension and sex of the participants, in several cases reaching more than 30%. The model with the highest explanatory power is the one obtained for men’s emotional attention. Likewise, these models include different predictors, although the most important predictors for each dimension of emotional intelligence are the other dimensions of emotional intelligence and mood modification social network addiction.

## Figures and Tables

**Table 1:** Descriptive statistics of the continuous research variables.

	Total (n = 411)	Men (n = 128)	Women (n = 283)	T-Student		Cohen’s d
				t	*p*-Value	
Age	20.47 (1.89)	20.91 (1.76)	20.28 (1.92)	3.15	0.002	0.34
Weight (Kg)	64.09 (11.81)	75.32 (10.01)	59.01 (8.63)	16.85	≤0.001	1.79
Height (m)	168.11 (9.71)	178.66 (6.73)	163.34 (6.57)	21.72	≤0.001	2.31
Body mass index	22.56 (2.90)	23.58 (2.67)	22.11 (2.90)	4.90	≤0.001	0.52
No. social networks	7.15 (2.05)	7.05 (2.12)	7.20 (2.01)	−0.79	0.479	0.07
No. of social networks used most days of the week	3.87 (1.18)	4.01 (1.16)	3.81 (1.18)	1.61	0.107	0.17
Emotional attention	30.32 (6.08)	28.18 (5.96)	31.29 (5.89)	−4.94	≤0.001	0.52
Emotional clarity	28.18 (6.34)	29.03 (5.94)	27.79 (6.49)	1.85	0.066	0.20
Emotional repair	28.51 (6.02)	30.07 (5.44)	27.80 (6.14)	3.59	≤0.001	0.38
Time management social network addiction	15.59 (5.10)	15.02 (4.62)	15.84 (5.28)	−1.51	0.132	0.16
Mood modification social network addiction	7.51 (3.13)	7.08 (3.07)	7.71 (3.26)	−1.84	0.066	0.20
Relapse social network addiction	6.49 (3.13)	6.20 (2.97)	6.63 (3.20)	−1.30	0.194	0.14
Withdrawal social network addiction	5.57 (2.93)	5.27 (2.70)	5.71 (3.03)	−1.39	0.165	0.15
Conflict social network addiction	5.51 (2.70)	5.53 (2.73)	5.50 (2.69)	0.12	0.909	0.01
Moderate physical activity (minutes per week)	146.43 (253.04)	173.39 (234.69)	134.24 (260.41)	1.46	0.147	0.15
Vigorous physical activity (minutes per week)	263.03 (323.72)	401.05 (329.75)	200.60 (305.73)	6.06	≤0.001	0.64
Moderate–vigorous physical activity (minutes per week)	409.46 (464.88)	574.45 (443.04)	334.84 (455.93)	4.98	≤0.001	0.53

**Table 2:** Descriptive statistics of the levels of the use of social networks.

Social Network	Sex	Use 4–7 Day/Week		X^2^	*p*-Value	Cramer’s V
		Yes n (%)	No n (%)			
Facebook	Men	56 (43.75)	72 (56.25)	0.23	0.632	0.02
	Women	131 (46.29)	152 (53.71)			
	Total	187 (45.50)	224 (54.50)			
Snapchat	Men	34 (26.56)	94 (73.44)	11.52	≤0.001	0.17
	Women	125 (44.17)	158 (55.83)			
	Total	159 (38.69)	252 (61.31)			
Telegram	Men	80 (62.5)	48 (37.5)	20.41	≤0.001	0.22
	Women	109 (38.52)	174 (61.48)			
	Total	189 (45.99)	222 (54.01)			
X	Men	90 (70.31)	38 (29.69)	0.27	0.604	−0.03
	Women	206 (72.79)	77 (27.21)			
	Total	296 (72.02)	115 (27.98)			
Pinterest	Men	33 (25.78)	95 (74.22)	94.29	≤0.001	−0.48
	Women	216 (76.33)	67 (23.67)			
	Total	249 (60.58)	162 (39.42)			
LinkedIn	Men	21 (16.41)	107 (83.59)	2.30	0.130	0.15
	Women	65 (22.97)	218 (77.03)			
	Total	86 (20.92)	325 (79.08)			
TikTok	Men	104 (81.25)	24 (18.75)	2.57	0.109	−0.08
	Women	247 (87.28)	36 (12.72)			
	Total	351 (85.40)	60 (14.60)			
Instagram	Men	125 (97.66)	3 (2.34)	1.01	0.315	−0.05
	Women	280 (98.94)	3 (1.06)			
	Total	405 (98.54)	6 (1.46)			
Twitch	Men	93 (72.66)	35 (27.34)	71.24	≤0.001	0.42
	Women	80 (28.27)	203 (71.73)			
	Total	173 (42.09)	238 (57.91)			
YouTube	Men	126 (98.44)	2 (1.56)	0.89	0.347	0.05
	Women	274 (96.82)	9 (3.18)			
	Total	400 (97.32)	11 (2.68)			
WhatsApp	Men	128 (100)	0 (0)	-	-	-
	Women	283 (100)	0 (0)			
	Total	411 (100)	0 (0)			
BeReal	Men	6 (4.69)	122 (95.31)	0.84	0.359	−0.05
	Women	20 (7.07)	263 (92.93)			
	Total	26 (6.33)	385 (93.67)			
Discord	Men	6 (4.69)	122 (95.31)	7.32	0.007	0.13
	Women	2 (0.71)	281 (99.29)			
	Total	8 (1.95)	403 (98.05)			

**Table 3:** Regression results for the whole sample.

	Variables	β	SE	*p*-Value	VIF	R^2^	Adjusted R^2^	RSE	DF	F	D-W
Emotional attention	Emotional clarity	0.338	0.045	≤0.001	1.259	0.294	0.283	5.14	1;404	4.298 *	1.917
	Mood modification social network addiction	0.443	0.098	≤0.001	1.542						
	Sex	3.357	0.558	≤0.001	1.038						
	Emotional repair	0.108	0.048	0.026	1.300						
	Time management social network addiction	0.129	0.062	0.037	1.527						
	X use	−1.189	0.573	0.039	1.028						
	Constant	10.409									
Emotional clarity	Emotional repair	0.373	0.045	≤0.001	1.094	0.324	0.314	5.26	1;404	5.170 *	1.749
	Emotional attention	0.358	0.046	≤0.001	1.176						
	Relapse social network addiction	−0.287	0.098	0.003	1.392						
	Conflict social network addiction	−0.286	0.113	0.012	1.377						
	Sex	−1.361	0.593	0.022	1.123						
	X use	1.323	0.582	0.024	1.016						
	Constant	10.677									
Emotional repair	Emotional clarity	0.364	0.046	≤0.001	1.247	0.247	0.238	5.26	1;405	5.244 *	1.829
	Sex	−2.062	0.586	≤0.001	1.097						
	Mood modification social network addiction	−0.388	0.099	≤0.001	1.508						
	Conflict social network addiction	0.308	0.100	0.002	1.444						
	Emotional attention	0.115	0.050	0.023	1.374						
	Constant	17.119									

*p* ≤ 0.05 (*). The value 0 for sex corresponds to males and 1 for females. The value 0 for the use of X corresponds to not using it most days of the week and 1 to using it (4–7 days/week).

**Table 4:** Regression results for male.

	Variables	β	SE	*p*-Value	VIF	R^2^	Adjusted R^2^	RSE	DF	F	D-W
Emotional attention	Mood modification social network addiction	0.680	0.178	≤0.001	1.655	0.390	0.354	4.790	1;403	5.754 *	2.061
	Emotional clarity	0.345	0.075	≤0.001	1.109						
	X use	−2.259	0.983	0.023	1.125						
	Facebook use	2.407	0.872	0.007	1.045						
	Instagram use	−7.106	2.924	0.017	1.092						
	Withdrawal social network addiction	0.535	0.186	0.005	1.697						
	Conflict social network addiction	−0.480	0.200	0.018	1.588						
	Constant	12.039									
Emotional clarity	Emotional repair	0.383	0.080	≤0.001	1.037	0.363	0.337	4.84	1;405	5.181 *	1.893
	YouTube use	−12.736	3.613	≤0.001	1.099						
	Relapse social network addiction	−0.566	0.150	≤0.001	1.076						
	Emotional attention	0.233	0.078	0.003	1.183						
	Vigorous physical activity	0.003	0.001	0.025	1.009						
	Constant	13.423									
Emotional repair	Emotional clarity	0.383	0.074	≤0.001	1.016	0.197	0.184	4.916	1;408	6.845 **	1.825
	Social network use	−0.992	0.379	0.010	1.016						
	Constant	22.916									

*p* ≤ 0.05 (*); *p* ≤ 0.01 (**). The value 0 for the use of X, Facebook, Instagram and YouTube corresponds to not using it most days of the week and 1 to using it (4–7 days/week). The variable social network use corresponds to the number of social networks used on most days of the week (4–7 days).

**Table 5:** Regression results for female.

	Variables	β	SE	*p*-Value	VIF	R^2^	Adjusted R^2^	RSE	DF	F	D-W
Emotional attention	Emotional clarity	0.320	0.053	≤0.001	1.245	0.230	0.221	5.19	1;407	4.304 *	1.846
	Mood modification social network addiction	0.482	0.095	≤0.001	1.011						
	Emotional repair	0.117	0.056	0.039	1.253						
	Constant	15.433									
Emotional clarity	Emotional repair	0.379	0.054	≤0.001	1.070	0.334	0.324	5.34	1;406	9.648 *	1.665
	Emotional attention	0.362	0.056	≤0.001	1.092						
	Relapse social network addiction	−0.339	0.101	≤0.001	1.030						
	X use	2.225	0.716	0.002	1.010						
	Constant	7.577									
Emotional repair	Emotional clarity	0.420	0.051	≤0.001	1.000	0.197	0.194	5.51	1;409	68.751 ***	1.857
	Constant	16.145									

*p* ≤ 0.05 (*); *p* ≤ 0.001 (***). The value 0 for the use of X corresponds to not using it most days of the week and 1 to using it (4–7 days/week).

## Data Availability

The data used to support the findings of the current study are available from the corresponding author upon request.

## Supplementary Materials

The following supporting information can be downloaded at https://www.mdpi.com/article/10.3390/ejihpe15070123/s1. Table S1. Correlation results for the whole sample; Table S2. Correlation results for male; Table S3. Correlation results for female.
