# Supplementary material for: Emotional Intelligence in Gen Z Teaching Undergraduates: The Impact of Physical Activity and Biopsychosocial Factors
Source: Eur J Investig Health Psychol Educ. 2025 Jul 4;15(7):123. doi: 10.3390/ejihpe15070123 (PMC12293475; doi:10.3390/ejihpe15070123)
Supplement: Supplementary file 1 [file ejihpe-15-00123-s001.zip › ejihpe-3555062-supplementary.pdf]

European Journal of
Investigation in Health, Psychology and Education

MDPI

Emotional Intelligence in Gen Z Teaching Undergraduates: The Impact of Physical Activity and Biopsychosocial Factors

Daniel Sanz-Martín [1,*], Rafael Francisco Caracuel-Cáliz [2,3,4], José Manuel Alonso-Vargas [5] and Irwin A. Ramírez-Granizo [5]

Supplementary Materials

Table S1. Correlation results for the whole sample.

	Age	Weight	Height	BMI	No. SN	No. SN-md	EA	EC	ER	TM-SNA	MM-SNA	R-SNA	W-SNA	C-SNA	MPA	VPA	MVPA
Age	-	**0.146**	**0.145**	0.074	**0.107**	-0.040	0.008	**0.129**	0.086	**-0.180**	**-0.147**	**-0.117**	**-0.183**	-0.037	0.071	**0.110**	**0.115**
Weight		-	**0.716**	**0.766**	0.062	0.092	**-0.208**	-0.026	0.044	0.059	0.046	0.029	0.004	0.010	0.050	**0.206**	**0.171**
Height			-	**0.109**	-0.063	0.015	**-0.209**	0.032	**0.102**	-0.035	-0.010	-0.045	0.015	-0.019	0.057	**0.215**	**0.181**
BMI				-	**0.139**	**0.119**	**-0.101**	-0.075	-0.038	**0.118**	0.080	0.088	-0.008	0.038	0.021	0.096	0.078
No. SN					-	**0.431**	0.077	-0.078	**-0.113**	**0.193**	**0.137**	0.083	**0.116**	0.070	-0.020	-0.024	-0.028
No. SN- md						-	0.078	0.042	-0.026	**0.144**	**0.153**	-0.001	**0.127**	0.054	-0.029	-0.046	-0.048
EA							-	**0.328**	**0.177**	**0.232**	**0.272**	**0.187**	**0.104**	**0.147**	0.009	-0.041	-0.024
EC								-	**0.437**	**-0.114**	**-0.123**	**-0.151**	-0.093	**-0.153**	0.035	0.096	0.086
ER									-	-0.010	**-0.150**	0.002	-0.086	-0.016	0.013	**0.104**	0.080
TM-SNA										-	**0.572**	**0.594**	**0.481**	**0.479**	-0.038	0.022	-0.005
MM-SNA											-	**0.542**	**0.468**	**0.452**	-0.042	-0.063	-0.067
R-SNA												-	**0.467**	**0.517**	0.010	0.037	0.031
W-SNA													-	**0.478**	0.027	0.010	0.022
C-SNA														-	-0.013	-0.014	-0.012
MPA															-	**0.289**	**0.745**
VPA																-	**0.853**
MVPA																	-

Note. Body mass index (BMI); SN (social network); SN-md (social networks used most days of the week); emotional attention (EA); emotional clarity (EC); emotional repair (ER); time management social network addiction (TM-SNA); mood modification social network addiction (MM-SNA); relapse social network addiction (R-SNA); withdrawal social network addiction (W-SNA); conflict social network addiction (C-SNA); moderate physical activity (MPA); vigorous physical activity (VPA); moderate–vigorous physical activity (MVPA); $p \leq 0.05$ (bold text format).

 https://doi.org/10.3390/ejihpe15070123

Table S2. Correlation results for males.

	Age	Weight	Height	BMI	No. SN	No. SN-md	EA	EC	ER	TM-SNA	MM-SNA	R-SNA	W-SNA	C-SNA	MPA	VPA	MVPA
Age	-	0.080	-0.009	0.099	0.164	-0.100	-0.041	-0.036	0.070	-0.041	-0.060	-0.028	-0.163	0.074	0.101	0.048	0.125
Weight		-	**0.491**	**0.824**	0.110	-0.054	-0.102	-0.057	-0.050	0.076	0.027	0.032	0.097	-0.086	-0.072	-0.008	-0.044
Height			-	-0.085	-0.155	-0.159	-0.100	-0.029	-0.033	-0.010	0.015	-0.011	0.011	-0.095	**-0.205**	-0.119	**-0.195**
BMI				-	**0.211**	0.048	-0.052	-0.040	-0.032	0.090	0.018	0.040	0.104	-0.037	0.052	0.072	0.080
No. SN					-	**0.477**	0.133	0.019	-0.159	**0.293**	**0.174**	0.066	0.156	0.131	0.027	-0.014	0.004
No. SN- md						-	0.147	0.127	-0.158	0.123	0.130	-0.042	**0.174**	0.101	0.039	-0.012	0.012
EA							-	**0.295**	**0.174**	**0.300**	**0.322**	**0.249**	0.033	**0.211**	0.090	-0.026	0.029
EC								-	**0.391**	**-0.190**	**-0.244**	**-0.197**	**-0.196**	**-0.236**	0.031	0.163	0.135
ER									-	-0.169	**-0.235**	0.004	-0.155	-0.129	-0.028	0.020	0.001
TM-SNA										-	**0.581**	**0.550**	**0.530**	**0.482**	0.058	-0.048	-0.004
MM-SNA											-	**0.552**	**0.502**	**0.537**	-0.027	**-0.205**	-0.162
R-SNA												-	**0.546**	**0.403**	0.028	-0.063	-0.031
W-SNA													-	**0.502**	0.046	-0.056	-0.016
C-SNA														-	-0.032	-0.138	-0.116
MPA															-	**0.255**	**0.714**
VPA																-	**0.859**
MVPA																	-

Note. Body mass index (BMI); SN (social network); SN-md (social networks used most days of the week); emotional attention (EA); emotional clarity (EC); emotional repair (ER); time management social network addiction (TM-SNA); mood modification social network addiction (MM-SNA); relapse social network addiction (R-SNA); withdrawal social network addiction (W-SNA); conflict social network addiction (C-SNA); moderate physical activity (MPA); vigorous physical activity (VPA); moderate–vigorous physical activity (MVPA); $p \leq 0.05$ (bold text format).

Table S3. Correlation results for female.

	Age	Weight	Height	BMI	No. SN	No. SN-md	EA	EC	ER	TM-SNA	MM-SNA	R-SNA	W-SNA	C-SNA	MPA	VPA	MVPA
Age	-	0.056	0.072	0.016	0.092	-0.034	0.083	**0.175**	0.058	**-0.218**	**-0.165**	**-0.140**	**-0.179**	-0.086	0.046	0.057	0.065
Weight		-	**0.464**	**0.832**	0.111	0.108	-0.061	**-0.137**	-0.109	**0.170**	**0.190**	**0.120**	0.048	0.057	0.043	0.050	0.058
Height			-	0.099	-0.007	-0.021	-0.033	-0.061	-0.041	0.044	0.112	0.009	**0.130**	-0.006	0.094	0.070	0.101
BMI				-	**0.126**	**0.127**	-0.047	**-0.122**	-0.001	**0.158**	**0.139**	**0.132**	-0.027	0.070	-0.014	0.012	0.000
No. SN					-	**0.416**	0.040	-0.116	-0.087	**0.150**	**0.118**	0.088	0.096	0.042	-0.037	-0.015	-0.003
No. SN- md						-	0.079	-0.093	0.006	**0.162**	**0.174**	0.023	**0.118**	0.033	-0.064	-0.019	-0.104
EA							-	**0.389**	**0.252**	**0.191**	**0.232**	**0.147**	0.114	**0.126**	0.001	0.056	0.038
EC								-	**0.443**	-0.078	-0.064	**-0.126**	-0-048	**-0.120**	0.028	0.035	0.040
ER									-	0.064	-0.100	0.017	-0.047	0.028	0.010	0.073	0.055
TM-SNA										-	**0.565**	**0.607**	**0.461**	**0.482**	-0.065	0.084	0.019
MM-SNA											-	**0.535**	**0.451**	**0.420**	-0.039	0.035	0.001
R-SNA												-	**0.435**	**0.568**	0.010	0.111	0.080
W-SNA													-	**0.471**	0.027	0.068	0.061
C-SNA														-	-0.006	0.042	0.025
MPA															-	**0.293**	**0.767**
VPA																-	**0.838**
MVPA																	-

Note. Body Mass Index (BMI); SN (Socia Network); SN-md (social networks used most days of the week); Emotional attention (EA); emotional clarity (EC); emotional repair (ER); Time-management social network addiction (TM-SNA); mood modification social network addiction (MM-SNA); relapse social network addiction (R-SNA); withdrawal social network addiction (W-SNA); conflict social network addiction (C-SNA); moderate physical activity (MPA); vigorous physical activity (VPA); moderate-vigorous physical activity (MVPA); $p \leq 0.05$ (bold text format).
